# Transcanal cochleostomy in cochlear implant surgery: long-term results of a cohort study

**DOI:** 10.1590/S1808-86942012000200018

**Published:** 2015-10-20

**Authors:** Michelle Lavinsky-Wolff, Luiz Lavinsky, Celso Dall'Igna, Joel Lavinsky, Ênio Setogutti, Manoela Chitolina Viletti

**Affiliations:** a Mestrado pela Universidade Federal do Rio Grande do Sul. Doutorado em andamento pela Universidade Federal do Rio Grande do Sul (Otorrinolaringologista do Hospital de Clínicas de Porto Alegre); b Doutorado e Pós-doutorado em Otorrinolaringologia (Professor do Departamento de Otorrinolaringologia da Universidade Federal do Rio Grande do Sul); c Doutorado pela Universidade Federal do Rio Grande do Sul (Professsor da Departamento de Otorrinolaringologia da Universidade Federal do Rio Grande do Sul); d Mestrado em andamento pela Universidade Federal do Rio Grande do Sul (Otorrinolaringologista); e Especialista em Ragiologia pela Sociedade Brasileira de Radiologia (Chefe do Departamento de Ressonância Nuclear Magnética do Serviço de Investigação Diagnóstica SIDI); f Médica formada pela Universidade Federal do Rio Grande do Sul (Médica Residente do Hospital de Clínicas de Porto Alegre). Universidade Federal do Rio Grande do Sul/Hospital de Clínicas de Porto Alegre

**Keywords:** auditory threshold, postoperative complications, cochlear implantation, otorhinolaryngologic surgical procedures

## Abstract

The combined approach technique (CAT) is a variation of the classical the mastoidectomy-posterior tympanotomy technique (MPTA) that combines a transcanal approach to cochleostomy with a reduced posterior tympanotomy for insertion of electrodes.

**Aim:**

To compare and evaluate long-term safety and effectiveness outcomes obtained with the CAT and with MPTA approach in patients submitted to cochlear implant (CI) surgery. Design: series study.

**Methods:**

Patients who underwent CI using CAT or MPTA at a Brazilian center were followed in a cohort study. Main outcomes were complications, audiometric performance and radiological evaluation of electrode position.

**Results:**

Fourty-four patients were implanted using CAT and 31 MPTA. There were no cases of facial nerve paralysis, mastoiditis, cholesteatoma or cerebrospinal fluid leaks after 3.4±1.0 years. Radiological evaluation of electrode position revealed that the median number of electrodes outside the cochlea was 0 in CAT and 3 in MPTA groups (*p* < 0.001). There were no differences between both surgical approaches in terms of mean pure-tone thresholds with CI at all frequencies.

**Conclusion:**

Long-term follow-up data showed that the transcanal route to cochleostomy, combined with a reduced posterior tympanotomy, is a safe alternative approach in cochlear implant surgery, with no related major complications and fewer cases of electrode migration when compared with the MPTA. These findings encourage the use of the transcanal route to cochleostomy as an alternative approach option.

## INTRODUCTION

The classic mastoidectomy-posterior tympanotomy approach (MPTA) for cochlear implantation (CI) was initially proposed by William House in 1961^1^, ^2^, and few changes have been made to the technique since then. The approach consists of a mastoidectomy followed by posterior tympanotomy. Cochleostomy is performed through the facial recess^1^, ^2^, and the facial nerve and chorda tympani are used as landmarks to demarcate the facial recess as a route of penetration into the middle ear. Although the method is well established, drilling through a narrow recess may lead to injury of the facial nerve or chorda tympani^3^. In addition, access is usually narrow and at an angle that makes it difficult to perform cochleostomy in anterior regions of the basal turn of the cochlea^4^.

In search of simpler and safer procedures, some alternative approaches have been studied in recent years^4^, ^5^, ^6^, ^7^, ^8^, ^9^, ^10^, ^11^, ^12^. The transcanal route to cochleostomy has been elected as a convenient approach by some authors^6^, ^7^, ^10^, ^11^, since it provides a direct exposure of middle ear landmarks and the cochleostomy site.

The combined approach technique (CAT), previously described^10^, ^11^, ^13^, is a variation of the classical MPTA technique that combines a transcanal approach to cochleostomy with a reduced posterior tympanotomy for insertion of electrodes.

This study reports long-term complications and effectiveness outcomes of CI surgery using the transcanal route (CAT) and compares these outcomes with those observed in a contemporary cohort of patients who underwent CI using the traditional approach.

## PATIENTS AND METHODS

Patients eligible for this longitudinal, observational, comparative study (cohort study) had severe or profound bilateral hearing loss, did not benefit from external hearing aids, and underwent CI consecutively from May 2003 to December 2006. A strict, standardized preoperative assessment consisting of clinical history, complete auditory tests, and psychological and social evaluations was carried out with all patients. Patients included in the study were consecutively scheduled to perform CI with one of two surgeons of the Cochlear Implant Program. The senior author performed all CAT surgeries, and all MPTA surgeries were performed by another experienced otologic surgeon of the Cochlear Implant Program. Patients were grouped according to the surgical approach used, MPTA or CAT. The implants used were Nucleus 24M and 24R and Contour (CochlearT, Lane Cove, Australia).

The study was conducted in accordance with the ethical principles of the Declaration of Helsinki, and was approved by the local Ethics Committee (08–005).

### Surgical protocol

#### Description of CAT surgeries (transcanal approach)

CAT is a variation of the classic MPTA. The initial steps are the same as in MPTA (skin incision, posterior periosteal flap elevation, creation of a posterior pouch and bony well for cochlear implant body fixation). The main additional modifications are the following: 1) development of a classic tympanomeatal flap; 2) transcanal exposure of middle ear structures; 3) small simple mastoidectomy and atticotomy with identification of the usual landmarks (antrum, horizontal semicircular canal, short process of the incus and the *fossa incudis* and posterior external auditory canal); 4) reduced posterior tympanotomy with exposure of the incudostapedial joint, generating a small but sufficient opening for subsequent insertion of the electrodes; 5) cochleostomy using the transcanal approach using the classic *scala tympani* cochleostomy site, inferior and anterior to the round window membrane; 6) insertion of the electrodes by small posterior tympanostomy and transcanal cochleostomy; 7) transcanal cochleostomy packing with connective tissue.

#### Description of MPTA surgeries

These surgeries were performed using the classic approach. Main steps were retroauricular approach, simple mastoidectomy, and posterior tympanotomy. Facial recess was opened using facial nerve and chorda tympani as landmarks to demarcate the route of penetration into the middle ear. *Scala tympani* cochleostomy was performed through the facial recess. Cochleostomy location was the same used in patients submitted to CAT surgery.

### Outcomes

Main outcomes assessed were intra- and postoperative complications, audiometric performance with CI, and radiological evaluation of electrode position.

Patient records were reviewed using a standardized protocol to obtain the following preoperative data: sex, age at implantation, duration of deafness, etiology of hearing loss, prelingual or postlingual deafness, previous experience with conventional hearing aids, spoken language skills, side of implantation, implant type and hearing thresholds before surgery.

Patients were prospectively evaluated from July 2007 to August 2008 during their routine follow-up visits. Investigators called all patients who missed regular visits to schedule a new appointment. All investigators signed a confidentiality agreement to preserve the secrecy of the information obtained from the review of patient records. This study was approved by the Ethics Committee at the institution where it was conducted (08–005).

### Intra- and postoperative complications

Patients' records were reviewed from May 2003 to June 2007 using a standardized protocol to assess intra- and postoperative complications. From July 2007 on, complications were prospectively evaluated during routine follow-up visits.

### Postoperative audiometric evaluation with CI

Postoperative audiograms were performed to test responses to pure tones between 500 and 4,000 Hz and open-set word and sentence recognition. Maximum output for 500 to 4,000 Hz frequencies was less than 80-dB hearing level (dB HL). Any response reported as vibrotactile or questionably vibrotactile was classified as no response. Pure tone averages (PTAs) were calculated for 500, 1,000 and 2,000 Hz frequencies^14^. For speech testing, subjects were seated in a sound-treated room with the CI adjusted to the user's most comfortable listening setting.

### Radiological evaluation of electrode position

Plain mastoid radiographs were taken in Stenver's and transorbital views to assess electrode position in the cochlea. Films were interpreted by an experienced radiologist blinded to the surgical approach used and to patient clinical performance. The counted rings (electrodes) were classified as outside the cochlea when they lay laterally to the optic capsule^15^. Since all patients had had electrodes fully inserted during the surgical procedure, electrodes outside the cochlea were considered as migrations. Electrode migration was classified as follows: 1) none, if all electrodes were inside the cochlea; 2) mild, if one or two electrodes were outside the cochlea; 3) moderate, if three to five electrodes were outside the cochlea; and 4) severe, if six or more electrodes were outside the cochlea.

### Statistical analysis

Sample size was calculated to analyze the association between surgical approach and postoperative audiometric mean pure-tone thresholds with CI. It was estimated that 7 patients would be needed in each group to detect a difference of 5 dB, at an alpha error of 5% and a beta error of 20%.

Data were reported as mean ± standard deviation, median, 25^th^ and 75^th^ percentiles, or percent. Statistical analyses were carried out using the Statistical Package for the Social Sciences version 14.0 (SPSS, Chicago, Illinois, United States of America). Results with a probability value of 0.05 or less were considered statistically significant.

The chi-square test was used for categorical variables, and the independent samples Mann-Whitney test for medians. The *t* test for independent samples was used for comparisons of means. Correlations were used to analyze the number of electrodes outside the cochlea, speech recognition scores and mean thresholds at each frequency.

## RESULTS

### Baseline characteristics

Seventy-five patients were included in the study; 44 were implanted using CAT, and 31, MPTA. Thirty-eight were male (51%); 26 (59%) in the CAT group and 12 (39%) in the MPTA group (p = 0.13). Age at implantation ranged from 1.9 to 69 years (median 8.4 years). Table 1 shows the baseline characteristics of all patients who underwent implantation.

**Table 1:** Baseline characteristics of 75 patients submitted to two approaches for cochlear implantation.

	CAT	MPTA	All
	(n = 44)	(n = 31)	(n = 75)
Age at implantation (years)			

Minimum	4.0	1.9	1.9
25^th^ percentile	5.0	6.0	5.3
50^th^ percentile (median)	7.8	9.8	8.4
75^th^ percentile	36.0	31.1	34.5
Maximum	60.8	68.8	68.8

Duration of deafness before CI (years)			

25^th^ percentile	4.1	4.1	4.1
50^th^ percentile (median)	6.0	6.8	6.2
75^th^ percentile	10.0	11.8	10.5
Prelingual deafness, n (%)	30 (68.1)	20 (64.5)	50 (66.6)
Previous experience with conventional hearing AIDS, n (%)	35 (79.5)	25 (80.6)	60 (80.0)
Spoken language, n (%)	18 (40.9)	18 (58.0)	36 (48.0)
Regular schooling, n (%)	22 (50.0)	21 (67.7)	43 (75.4)

CAT: combined approach technique; MPTA: mastoidectomy-posterior tympanotomy approach; CI: cochlear implantation. *p* > 0.05 for all variables.

### Complications

Table 2 presents the complications observed in both groups. Mean follow-up for this outcome was 3.4 ± 1.0 years (range: 1.5 to 5.2 years). There were no cases of facial nerve paralysis, mastoiditis, cholesteatoma, or cerebrospinal fluid leaks. A case of wound infection and breakdown and implant partial extrusion in the CAT group occurred 28 months after implantation.

**Table 2:** Complications in 75 cochlear implant patients according to surgical approach*.

Complications	CAT, n (%)	MPTA, n (%)
	(n = 44)	(n = 31)
Wound breakdown and receiver-stimulator partial extrusion+	1 (2.0)	0
Perforated eardrum	0	1 (3.0)
Temporary balance disturbance	2 (4.0)	1 (3.0)
Wound hyperemia	1 (2.0)	0
External auditory canal granuloma, first postoperative week	4 (9.0)	0
Skin wound over receiver-stimulator	1 (2.0)	1 (3.0)
Retroauricular keloid	1 (2.0)	0
Electrode migration**§		
None	18 (62.0)	3 (12.0)
Mild (1–2)	7 (24.0)	8 (32.0)
Moderate (3–5)	4 (14.0)	9 (36.0)
Severe (≥ 6)	0	5 (20.0)

CAT: combined approach technique; MPTA: mastoidectomy-posterior tympanotomy approach.

* There were no cases of facial nerve paralysis, mastoiditis, cholesteatoma or cerebrospinal fluid leaks.

+ Complication occurred 28 months postoperatively.

** Number of patients in this complication category was 29 in CAT group and 25 in MPTA group.

§ *p* < 0.001.

### Electrode migration

Radiological evaluation was performed at a mean of 34±12 months postoperatively (range: 11 to 53 months). Patients who missed scheduled radiological evaluations (n = 19), a patient with extruded cochlear implant (n = 1), and one case of bad quality plain film (n = 1) were excluded from this analysis.

The median number of electrodes outside the cochlea was 0 in the CAT group (25^th^ percentile = 0; 75^th^ percentile = 1) versus 3 in the MPTA group (25^th^ percentile = 1; 75^th^ percentile = 4; *p* < 0.001). Cases classified as none, mild, moderate or severe migration are shown in Table 2. Number of electrodes outside the cochlea was not correlated with mean thresholds at 500 to 4,000 Hz, speech recognition scores and PTAs with CI (*p* > 0.9 for all tested variables).

### Audiometric evaluation with CI

Mean follow-up was 28 ± 13 months. Mean postoperative pure-tone thresholds obtained with CI at different frequencies are presented in Table 3. There were no differences between surgical approaches in terms of mean pure-tone thresholds in any frequency (*p* > 0.05 for 500 to 4,000 Hz). PTA, calculated as the mean of patient thresholds at 500, 1,000 and 2,000 Hz, was 42.0 ± 13.8 dB for patients in the CAT group and 38.5±10.6 dB for MPTA patients (*p* = 0.252). Median dissyllabic word recognition score without lip reading was 30% in the CAT group (25^th^ percentile = 0; 75^th^ percentile = 80%) and 36% in the MPTA group (25^th^ percentile = 0; 75^th^ percentile = 80%; *p* > 0.05).

**Table 3:** Mean postoperative audiometric pure-tone thresholds with cochlear implant according to surgical approach*+.

	Mean threshold (dB)	
	CAT (n = 44)	MPTA (n = 31)
500 Hz	43.2±12.1	40.0±10.1
1,000 Hz	41.3±13.8	37.0±11.6
2,000 Hz	41.5±16.9	38.4±11.6
4,000 Hz	45.7±19.9	41.3±15.5

CAT: combined approach technique; MPTA: mastoidectomy-posterior tympanotomy approach.

* Maximum audiometer output +5 dB was used to include patients with no response in any frequency.

+ Mean follow-up: 2.3±1.0 years. *p* > 0.05 for all frequencies.

## DISCUSSION

The transcanal route has the advantage of providing wide and direct exposure of middle ear structures and cochleostomy site. The drill can be positioned at an angle that facilitates the procedure and avoids proximity to the facial nerve. Wide exposure of the middle ear with better visualization of the promontory also results in easier drilling of the cochleostomy and better control of electrode insertion into the *scala tympani*^4^. As the cochleostomy is performed directly through the middle ear, CAT requires a reduced posterior tympanotomy, large enough for the insertion of electrodes, avoiding a larger opening of the facial recess. The drilling can thus be conducted at a comfortable distance from the facial nerve, simplifying this surgical step.

In short, the main advantages of the proposed CAT are its simplicity, resulting from the easily performed cochleostomy, the small posterior tympanotomy, and the direct visualization of electrodes during insertion. In our experience, these advantages resulted in reduced surgical time, even considering the additional minutes spent with preparation of the tympanomeatal flap^10^, ^11^, ^13^.

However, the question that motivated this paper was whether these interesting modifications to the usual approach for CI could produce any unfavorable clinical outcome. Therefore, our study compared long-term clinical effectiveness and complications associated with both CAT and MPTA. This is one of the few comparative long-term follow-up clinical studies designed to compare an alternative surgical approach to CI with the conventional approach. Although this trial was not randomized, both groups had similar characteristics, and there were no significant differences in age at implantation, sex, language skills, schooling, years of deafness before surgery, and experience with CI by the two surgeons involved.

The analysis of long-term follow-up data revealed that both groups had equally satisfactory audiometric and speech recognition results. Moreover, this clinical performance with CI should improve during the first 10 years post-implantation, as demonstrated in previous studies^16^, ^17^.

Major complications, such as facial nerve paralysis, meningitis, cholesteatoma and cerebrospinal fluid leaks, did not occur in either the CAT or the MPTA groups. Studies conducted in major centers have also reported an overall low incidence (0.3% to 3.0%) of major complications in CI using MPTA^18^. Although rare, complications such as facial nerve paralysis and meningitis can be devastating. Our long-term evaluation data suggest that CAT was at least as safe as the traditional access technique.

Device migration is the movement of CI away from its original site. Some authors classify it as a major complication^19^. To avoid electrode migration, several techniques have been developed, such as packing cochleostomy tightly with tissue, placing a loop of electrode cable against the tegmen mastoideum, and aligning the electrode cable with the cochleostomy to reduce the force of migration^20^. Cohen & Kuzma^21^ have developed a titanium clip to secure the electrode to the incus buttress, and Balkany & Telischi^22^ have described a technique to secure the electrode within a slot in the incus buttress (split-bridge technique).

Studies report a low overall rate (about 1%) of electrode migration^15^, but some authors have found that it is a leading cause of reimplantation, second only to device failure^20^, ^23^. A review of 3,773 cases in Latin America^24^ showed that full migration occurred in 13 cases (0.35%), most of them associated with ceramic implants (ClarionT, MED-ELT, 3MT).

In our study, radiological evaluation after a mean of 2.8 ± 1.0 years revealed that full migration occurred in 2% of the cases, all in the MPTA group. Our data demonstrated that the group undergoing CAT had significantly fewer cases of electrode migration than the MPTA group: 0% severe migration with CAT (≥ 6 electrodes outside cochlea) versus 20% with MPTA (*p* < 0.001). Although this difference might be attributed to differences in the technical skills of the two surgeons involved in the study rather than to the two surgical approaches, we believe that the long-term stability observed in CAT patients may be due to the fact that the transcanal approach provides easier access to the cochleostomy site and a favorable angle for cochleostomy drilling, electrode insertion, and tight packing with connective tissue under direct view. Also, the small posterior tympanotomy, packed with connective tissue and bone dust, contributes to this stability^25^.

Mobilization of the tympanomeatal flap, in the same way as in routine stapedectomy, did not result in postoperative infection in our patients. Other alternative techniques also involve mobilization of the tympanomeatal flap, with no increase in cases of postoperative infection^6^, ^7^. Minor complications, such as external auditory canal granuloma, occurred in four patients undergoing CAT during the first postoperative week, all of which had total resolution after topical antibiotic treatment.

## CONCLUSION

Long-term follow-up data showed that the transcanal route to cochleostomy combined to a reduced posterior tympanotomy (CAT) is a safe alternative approach in CI surgery, with no related major complications and fewer cases of electrode migration when compared with MPTA. Both approaches were equally effective in terms of postoperative hearing performance with CI. These findings encourage the use of the transcanal route to cochleostomy as an alternative approach option, as advocated by other authors previously.

## References

[1] House WF (1976). Cochlear implants. Ann Otol Rhinol Laryngol..

[2] House WF (1982). Surgical considerations in cochlear implantation. Ann Otol Rhinol Laryngol Suppl..

[3] Migirov L, Yakirevitch A, Kronenberg J (2006). Surgical and medical complications following cochlear implantation: comparison of two surgical approaches. ORL J Otorhinolaryngol Relat Spec..

[4] Goycoolea MV, Ribalta GL (2003). Exploratory tympanotomy: an integral part of cochlear implantation. Acta Otolaryngol..

[5] Colletti V, Fiorino FG, Carner M, Pacini L (1998). Basal turn cochleostomy via the middle fossa route for cochlear implant insertion. Am J Otol..

[6] Kronenberg J, Migirov L (2006). The suprameatal approach: an alternative surgical technique for cochlear implantation. Cochlear Implants Int..

[7] Kiratzidis T, Iliades T, Arnold W (2002). Veria operation. II. Surgical results from 101 cases. ORL J Otorhinolaryngol Relat Spec..

[8] Häusler R (2002). Cochlear implantation without mastoidectomy: the perica-nal electrode insertion technique. Acta Otolaryngol..

[9] Labadie RF, Noble JH, Dawant BM, Balachandran R, Majdani O, Fitzpatrick M (2008). Clinical validation of percutaneous cochlear implant surgery: initial report. Laryngoscope..

[10] Lavinsky L, Lavinsky M, Lavinsky L. (2006). Tratamento em otologia.

[11] Lavinsky L, Lavinsky M (2006). Combined approach technique to cochlear implantation. Otolaryngol Head Neck Surg..

[12] Slavutsky V, Nicenboim L (2009). Preliminary results in cochlear implant surgery without antromastoidectomy and with atrauma-tic electrode insertion: the endomeatal approach. Eur Arch Otorhinolaryngol..

[13] Lavinsky L, Lavinsky-Wolff M, Lavinsky J (2010). Transcanal cochleostomy in cochlear implantation: experience with 50 cases. Cochlear Implants Int..

[14] Balkany TJ, Connell SS, Hodges AV, Payne SL, Telischi FF, Eshraghi AA (2006). Conservation of residual acoustic hearing after cochlear implantation. Otol Neurotol..

[15] Roland JT, Fishman AJ, Waltzman SB, Alexiades G, Hoffman RA, Cohen NL (1998). Stability of the cochlear implant array in children. Laryngoscope..

[16] Beadle EA, McKinley DJ, Nikolopoulos TP, Brough J, O'Donoghue GM, Archbold SM (2005). Long-term functional outcomes and academic-occupational status in implanted children after 10 to 14 years of cochlear implant use. Otol Neurotol..

[17] Calmels MN, Saliba I, Wanna G, Cochard N, Fillaux J, Deguine O (2004). Speech perception and speech intelligibility in children after cochlear implantation. Int J Pediatr Otorhinolaryngol..

[18] Fayad JN, Wanna GB, Micheletto JN, Parisier SC (2003). Facial nerve paralysis following cochlear implant surgery. Laryngoscope..

[19] Kubo T, Matsuura S, Iwaki T (2005). Complications of cochlear implant surgery. Oper Tech Otolaryngol..

[20] Connell SS, Balkany TJ, Hodges AV, Telischi FF, Angeli SI, Eshraghi AA (2008). Electrode migration after cochlear implantation. Otol Neurotol..

[21] Cohen NL, Kuzma J (1995). Titanium clip for cochlear implant electrode fixation. Ann Otol Rhinol Laryngol Suppl..

[22] Balkany T, Telischi FF (1995). Fixation of electrode cable during cochlear implantation: the split bridge technique. Laryngoscope..

[23] Cullen RD, Fayad JN, Luxford WM, Buchman CA (2008). Revision of cochlear implant surgery in children. Otol Neurotol..

[24] Goycoolea MV (2005). Latin America Cochlear Implant Group. Latin American experience with the cochlear implant. Acta Otolaryngol..

[25] Lavinsky-Wolff M, Lavinsky L (2008). Análise comparativa de duas técnicas de implante coclear: estudo de coorte com resultados em longo prazo [dissertação]..

